# Excessive bone formation in a mouse model of ankylosing spondylitis is associated with decreases in Wnt pathway inhibitors

**DOI:** 10.1186/ar4096

**Published:** 2012-11-22

**Authors:** Katelin R Haynes, Allison R Pettit, Ran Duan, Hsu-Wen Tseng, Tibor T Glant, Matthew A Brown, Gethin P Thomas

**Affiliations:** 1 University of Queensland Diamantina Institute, Princess Alexandra Hospital, Ipswich Road, Brisbane, QLD 4101, Australia; 2 University of Queensland Centre for Clinical Research, Royal Brisbane & Women's Hospital Campus, Bowen Bridge Road, Herston, QLD, 4029, Australia; 3 Department of Orthopedic Surgery, Rush University Medical Center, 1653 West Congress Parkway, Chicago, IL-60612, USA

## Abstract

**Introduction:**

Ankylosing spondylitis (AS) is unique in its pathology where inflammation commences at the entheses before progressing to an osteoproliferative phenotype generating excessive bone formation that can result in joint fusion. The underlying mechanisms of this progression are poorly understood. Recent work has suggested that changes in Wnt signalling, a key bone regulatory pathway, may contribute to joint ankylosis in AS. Using the proteoglycan-induced spondylitis (PGISp) mouse model which displays spondylitis and eventual joint fusion following an initial inflammatory stimulus, we have characterised the structural and molecular changes that underlie disease progression.

**Methods:**

PGISp mice were characterised 12 weeks after initiation of inflammation using histology, immunohistochemistry (IHC) and expression profiling.

**Results:**

Inflammation initiated at the periphery of the intervertebral discs progressing to disc destruction followed by massively excessive cartilage and bone matrix formation, as demonstrated by toluidine blue staining and IHC for collagen type I and osteocalcin, leading to syndesmophyte formation. Expression levels of DKK1 and SOST, Wnt signalling inhibitors highly expressed in joints, were reduced by 49% and 63% respectively in the spine PGISp compared with control mice (P < 0.05) with SOST inhibition confirmed by IHC. Microarray profiling showed genes involved in inflammation and immune-regulation were altered. Further, a number of genes specifically involved in bone regulation including other members of the Wnt pathway were also dysregulated.

**Conclusions:**

This study implicates the Wnt pathway as a likely mediator of the mechanism by which inflammation induces bony ankylosis in spondyloarthritis, raising the potential that therapies targeting this pathway may be effective in preventing this process.

## Introduction

Ankylosing spondylitis (AS) displays a unique pathology in its progression from an initial inflammatory phase to an osteoproliferative/ankylosing phase, which can result in joint fusion [1]. The inflammatory phase has similarities with other inflammatory arthopathies such as rheumatoid arthritis (RA) with high levels of pro-inflammatory cytokine production and joint damage through osteoclast activity [2]. However, whereas the synovitis of RA is associated with joint erosion, while there is initial erosion in AS, the joint disease is primarily characterised by osteoproliferation and consequent ankylosis. There is considerable debate as to how the inflammation and osteoproliferation are linked, including whether the inflammation directly leads to the osteoproliferation, ceases before induction of bone formation, or whether the inflammatory and osteoproliferative phases are completely uncoupled [3]. The initial inflammation occurs in axial entheses, such as the spinal and sacroiliac ligament attachments, or sites of attachment of the annulus fibrosus outer fibres of the intervertebral discs (IVDs), progressing to osteoproliferation, squaring of the vertebrae and formation of syndesmophytes from the vertebral corners, which can eventually bridge leading to ankylosis. How this inflammation is initiated and how it progresses through to bone formation and eventual ankylosis is poorly understood.

A number of informative studies have characterised disease progression using radiography and magnetic resonance imaging (MRI) [4,5] but such modalities can only inform on gross structural changes. Elucidation of the cellular and molecular changes that contribute to disease progression requires tissue samples from disease sites. However, the difficulties in obtaining biopsy at axial skeletal sites means very few informative clinical samples are available. Animal models are thus a good option for examining detailed events occurring at axial disease sites. Although a number of animal models present some of the features similar to those seen in human disease, no mouse model as yet has proven to be a good model in which to study the progression from inflammation to ankylosis in the axial skeleton. Transgenic rats over-expressing the HLA-B27 and human β2-microglobulin have been shown to spontaneously display gut disease and peripheral and axial inflammatory arthritis [6], but ankylosis was only seen in rats with increased expression of β2-microglobulin, which coincided with reduced gut disease and unfolded protein response [7]. Two mouse models over-expressing TNF-α, either through a transgenic approach (hTNFtg) [8], or through increasing TNF mRNA stability by deleting the 3' ARE regulatory elements (TNFΔARE)[9], show systemic inflammation, gut disease and sacroiliitis but do not spontaneously develop ankylosis. Several mouse models have exhibited spontaneous ankylosing enthesopathy (ANKENT), including C57BL/10 [10] and DBA/1 [11] mice, but this has been limited to peripheral joints. The only inducible mouse model demonstrating axial ankylosis as well as a strong immune component is the proteoglycan (PG)-induced spondylitis model (PGISp). Disease is induced by injections of a human cartilage PG extract, and mimics many of the clinical features of the human disease, particularly axial inflammation and ankylosis stemming from an initial inflammatory stimulus [12,13].

The Wnt pathway has been established as a key regulatory pathway for the bone-forming cells, osteoblasts, stimulating both osteoblast proliferation and maturation [14]. During canonical Wnt signalling in bone, soluble Wnts bind to their Frizzled (Fzd) receptors and LRP4/5/6 co-receptors in a ternary complex at the cell surface, resulting in glycogen synthase kinase 3 (GSK-3β) inhibition, permitting β-catenin (β-cat) accumulation. Accumulated β-cat translocates into the nucleus and activates target gene transcription. In the absence of signalling, β-cat is phosphorylated, mainly by GSK3β, resulting in β-cat ubiquitination and proteasome-mediated degradation. In bone, Dickkopf-1 (DKK-1), sclerostin (SOST) and the secreted Fzd-related proteins are key inhibitors of the Wnt pathway, binding either to LRP4/5/6 inhibiting its interaction with the Wnt-Fzd complex, or associating with Wnts, blocking their participation in the pathway [14]. A number of recent papers have demonstrated changes in SOST and DKK1 in the hTNFtg mouse model and in AS patients, suggesting a role for this pathway in the osteoproliferation characteristic of AS [15-17].

We therefore sought to investigate whether SOST and DKK1 are dysregulated in the PGISp model, given it is a good experimental model for assessing cellular and molecular events involved in severe axial osteoproliferation and ankylosis as seen in AS. In the studies described here we have undertaken an in-depth morphological and molecular study to examine the nature of the spondylitis and identified molecular changes that might contribute to the joint changes in the PGISp model. As well as identifying changes in matrix component expression levels that underlie the excessive bone matrix formation seen, we also observed decreases in Wnt-signalling inhibitors that might promote increased bone formation contributing to ankylosis.

## Materials and methods

### Mouse model

The PGISp model has been described previously [12,13]. For the studies described here the model was established on the IL4^-/- ^background due to the reported increased penetrance [18]. Briefly, three-month old female mice were injected with 2 mg of human cartilage extract equivalent to 100 μg of PG core protein, together with 2 mg dimethyl dioctadecyl ammoniumbromide (Sigma, St. Louis, MO, USA). Three month-old female mice were injected three times at day 0, 21 and 42. Mice were collected at week 12 for the subsequent analyses described below. All mouse studies were carried out under the approval of the University of Queensland Animal Ethics Committee. For the histology studies four control and seven PGISp mice were analysed. For the gene expression analyses, seven control and six PGISP mice were analysed and of these, four control and four PGISp mice were used for the microarray study.

### Bone fixation/histology

Skeletons were collected and fixed in neutral buffered formalin and decalcified in 14% ethylenediaminetetraacetic acid (EDTA). Sections were stained with H&E or toluidine blue according to standard protocols. Gruber's stain has been described previously [19] with a combination of Weigert's haematoxylin, alcian blue and picrosirius red producing distinctive staining of collagen (red), PG (blue) and the cellular elements of the IVD. Severity of vertebral joint disease was scored as described previously [13]; score 1, enthesitis, inflammatory cell accumulation around the IVD and/or infiltration of the annulus fibrosus; score 2, < 50% absorption/erosion of the IVD; score 3, essentially complete resorption (> 50%) of the IVD; score 4, cartilaginous/bony ankylosis.

For immunohistochemistry (IHC), collagen type I (ColI) and osteocalcin (OCN) staining were performed as described in Chang *et al*. [20] and sclerostin (SOST) staining as in Walsh *et al*. [21].

### RNA extraction and microarray analysis

Whole spines from control and PGISp mice were flash-frozen on collection and stored at -80°C. Due to the requirements to preserve RNA integrity it was not possible to assess the activity of arthritis in the spines before RNA extraction, but only mice displaying severe peripheral arthritis were selected. RNA was extracted using Trizol (Life Technologies, Mulgrave, Victoria, Australia) as per the manufacturer's instructions as described previously [22], then cleaned using RNAeasy columns (Qiagen, Doncaster, Victoria, Australia). For microarray analysis cRNA was generated from 500 ng total RNA using the Illumina TotalPrep cRNA Amplification Kit and hybridised to Mouse Ref-8 Expression BeadChips (Illumina, San Diego, CA, USA). Array data were processed as described previously [23] using the Illumina GenomeStudio software and then transformed by variance stabilization transformation (VST) and normalized by robust spline normalization using Lumi [24]. Gene expression analysis was performed in BRB-ArrayTools [25]. Differentially expressed genes were identified by unpaired *t*-test with multivariate permutation correction. These data have been uploaded to the NCI GEO database [GEO: GSE41039].

### Gene Ontology analysis

The evaluation of the gene ontology (GO) classes that are differentially expressed between control and affected bones was performed using a functional class scoring analysis as described previously [26]. For each gene in a GO class, the *P*-value for comparing control and affected samples was computed. The set of *P*-values for a class was summarised by two summary statistics: (i) The log summary (LS) is the average of the log *P*-values for the genes in that class and (ii) the Kolmogorov-Smirnov summary (KS) is the Kolmogorov-Smirnov statistic computed on the *P*-values for the genes in that class. The statistical significance of the GO class containing n genes represented on the array was evaluated by computing the empirical distribution of these summary statistics in random samples of n genes. Functional class scoring is a more powerful method of identifying differentially expressed gene classes than the more common over-representation analysis or annotation of gene lists based on individually analysed genes. Efron-Tibshirani's gene set analysis (GSA) was also used, which uses maxmean statistics for assessing the significance of predefined gene sets and generates a direction of significant expression changes. GO analysis was performed using BRB-ArrayTools.

To examine a possible shift towards osteoblastic activity in the affected spine we generated an osteoblast gene list comprised of well-characterised genes known to be expressed in osteoblasts, which is not available in the standard GO analysis. The genes incorporated in this list were *Sp7*, *Ifitm5*, *Sost*, *Ostn*, *Runx2*, Osteocalcin (*Bglap*), Rankl (*Tnfrsf11b*), Rank (*Tnfsf11*), *Dmp1, Phex*, Alkaline phosphatase (*Alpl*), *Col1a1, Ibsp, Spp1, Pthr*1 and *Sparc*.

### Quantitative PCR

Both *Sost *and *Dkk1 *were expressed at background levels in the microarray study so we utilised quantitative real-time reverse-transcription PCR (qPCR) to accurately measure their transcription levels. RNA (1000 ng) was reverse-transcribed using both oligo-dT primers and random hexamers using the Bioline cDNA Synthesis Kit (Bioline, Alexandria, NSW, Australia) and expression was measured using Taqman assays (Life Technologies, Mulgrave, Victoria, Australia) for *Sost *and *Dkk1 *and *b-Actin *was used as a housekeeping gene to normalise expression.

Comparison of control and PGISP joints for histological scoring, expression levels of *Sost *and *Dkk1 *and SOST IHC were analysed using GraphPad Prism 5 (GraphPad Software Inc, La Jolla, CA, USA), using non-parametric Mann-Whitney tests. Data are expressed at the mean ± SD.

## Results

Inflammation followed a similar time course to that previously described for this model [18]. Initial peripheral inflammation in the knees and ankles, as well as the forelimbs, was evident after the second PG injection and peripheral inflammation increased over a further 8-week period before maximising at 12 weeks after the start date (data not shown). Our focus for this study was on the spondylitis, previously described in this model [13]. Mice were analysed 12 weeks (day 84) after the first PG injection (day 0) when both axial and peripheral disease was well-established.

**Figure 1:**
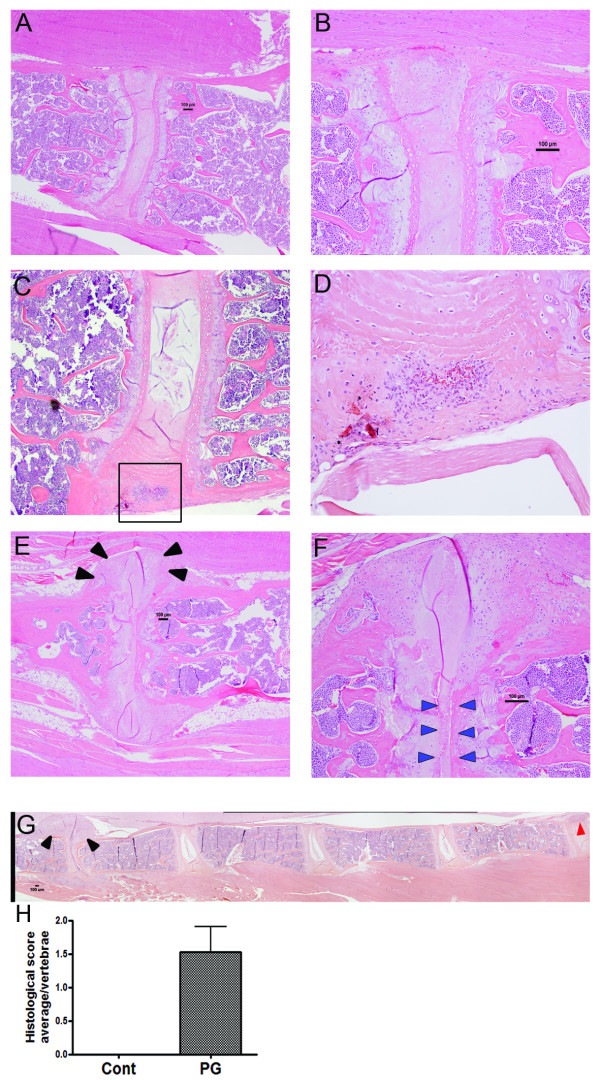
**Morphology of unaffected and affected sacral vertebrae in the proteoglycan-induced spondylitis (PGISp) mouse**. An unaffected sacral vertebral joint from a week-12 mouse shows an intact intervertebral disc (IVD) without inflammation; H&E staining ×40 (**A**) and ×200 (**B**). Early inflammation is evident in the IVD of PGISp mice 12 weeks after the first proteoglycan (PG) injection (boxed area); ×40 (**C**) and ×200 (**D**). Severely-affected sacral joint in week-12 PGISp show massive mesenchymal cell proliferation, excess matrix formation (black arrowheads) and almost complete IVD destruction (blue arrowheads); ×40 (**E**), ×100 (**F**). Inflammation and joint damage is also found in the spine and variable penetrance, a characteristic of AS, is also evident. Severely damaged (black arrowheads) and early-stage inflamed (red arrowhead) joints are found in close proximity to unaffected joints; ×40 (**G**). Histological scoring of the vertebrae indicates the degree of disease severity in the PGISp mice (**H**).

In the spine and sacral IVDs extensive inflammation and arthritis was found in all affected mice. Similar to human AS [27], not all vertebrae were affected and the severity of the arthritis also varied amongst different vertebrae in a single mouse. Figure 1A and 1B show an unaffected sacral joint in a mouse 12 weeks after the initial PG injection. H&E staining clearly shows an intact joint with no inflammatory cell invasion or damage. In an adjacent IVD, the early stages of inflammation can be seen in Figure 1C and 1D, with invasion of inflammatory cells. A severely affected joint is depicted in Figure 1E and 1F, where the initial inflammation has progressed to massively excessive tissue formation resembling the early stages of a syndesmophyte, which is associated with significant numbers of proliferative mesenchymal cells coupled with almost complete destruction of the IVD. Figure 1G further illustrates the variable nature of the lesions in a 12-week mouse spine with IVDs displaying early stage inflammation as well as severe damage adjacent to unaffected joints. To quantify the extent of disease in the mice the vertebrae were scored on a scale of 1 to 4 as described previously [13] with the average score per vertebrae per mouse being 1.5 ± 0.4 (Figure 1H). We also looked at mice up to 24 weeks after the first injection and they showed similar morphological changes to those at 12 weeks, except for greater penetrance, with most joints severely affected by frequent excessive tissue formation and bridging syndesmophyte formation (data not shown).

**Figure 2:**
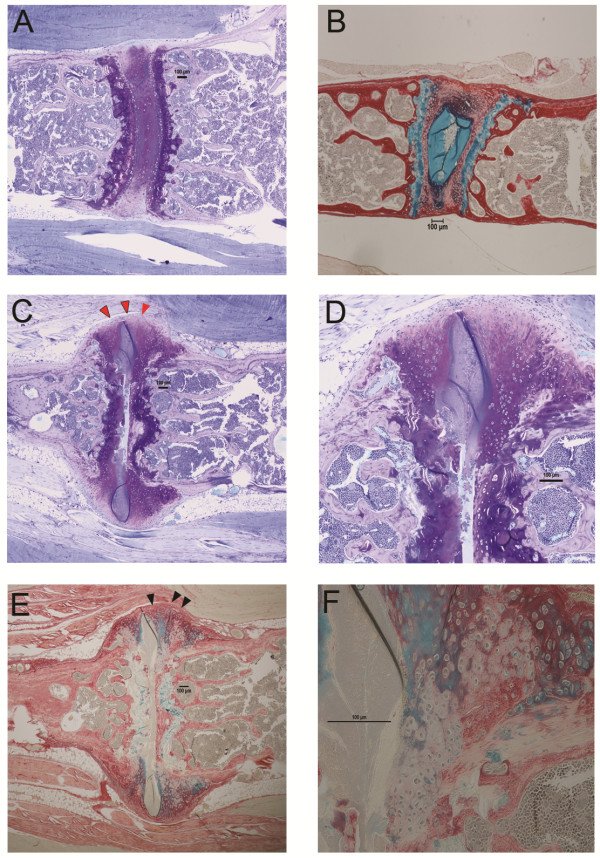
**Matrix changes in affected proteoglycan-induced spondylitis (PGISp) mouse vertebrae**. (**A**) Toluidine blue staining in unaffected week-12 control vertebrae shows clear staining in the vertebral growth plate cartilage and also in the nucleus pulposus (×40). Note the lack of staining in the bone. (**B**) Further tissue delineation is shown using Gruber's intervertebral disc (IVD) stain (×40). Collagen in the bone and annulus fibrosus is stained red, and proteoglycan (PG) in the cartilage and nucleus pulposus is stained blue. The cellular nature of the IVD is clearly visible. Toluidine blue staining of the same affected sacral joint shown in Figure 1 shows that the majority of the excess matrix laid down is PG-rich. However, note the fringes of this matrix stain negative for PG (red arrowheads); ×40 (**C**), ×100 (**D**). Gruber's IVD stain further illustrates the PG-rich nature of the excess matrix; ×40 (**E**), ×100 (boxed area in **F**). However the PG-negative fringes also stain positive for collagen (black arrowheads).

To further delineate the nature of the components of the excessive matrix formation shown in Figure 1 we used toluidine blue, which stains for PG, and Gruber's IVD staining, which differentiates between collagen (red) and PG (blue) [19], to delineate matrix components. In unaffected IVDs strong PG staining is seen in the cartilage endplates and in the nucleus pulposus (Figure 2A, B). Collagen staining clearly delineates the vertebral bone tissue but shows only faint staining in the IVD, representing the lower collagen content in this tissue. However, in affected joints no PG staining is evident within the disc due to extensive destruction, but extensive staining is seen in the excessive matrix laid down at axes of the joints (Figure 2C, D). The periphery of this matrix is low in PG but Gruber's stain shows it is positive for collagen staining (Figure 2E, F).

**Figure 3:**
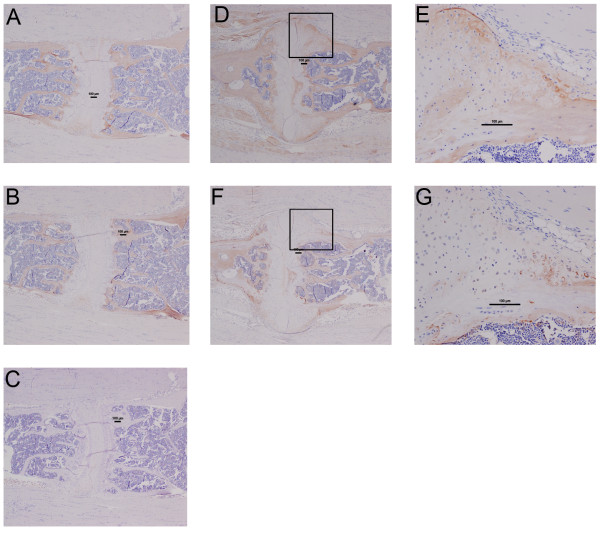
**Bone matrix proteins in affected proteoglycan-induced spondylitis (PGISp) mouse vertebrae**. In an unaffected sacral joint, collagen type I (**A) **(×40) and osteocalcin (**B**) (×40) immunohistochemical staining clearly delineate the bone compartment, only staining the vertebral bone. Collagen type I staining is evident at the fringes of the excess matrix in an affected joint however (D) (×40), (boxed area in **E**) (×200). This collagen matrix is only osteocalcin-positive at the outermost periphery (**F**) (×40), (boxed area in **G**) (×200,). Rabbit immunoglobulin (IgG)-negative control shows no background staining (**C**) (×40).

Figure 2 indicates the excess matrix underlying syndesmophyte formation was largely PG-rich, except at the periphery where the PG staining was less evident, with more apparent collagen positivity. Advanced AS frequently presents as fusion of the vertebral bodies due to bony matrix bridging across adjacent vertebrae. To further investigate the nature of the excess matrix formation in this model we used IHC to confirm the presence of the bone matrix components, Col1 and OCN in the affected tissue. In intact joints both Col1 I (Figure 3A) and OCN (Figure 3B) clearly delineate the vertebral bone but no staining is seen in the disc space. Similarly in affected discs, the vertebral bone is stained (Figure 3D and 3F) but some Col1 staining is also evident at the periphery of the syndesmophyte (Figure 3E) concurring with the Gruber's collagen staining in Figure 2E. However this matrix was largely negative for the more mature bone protein OCN, with slight staining at the periphery. The unaffected mature vertebral trabecular bone was strongly positive for OCN (Figure 3G).

**Figure 4:**
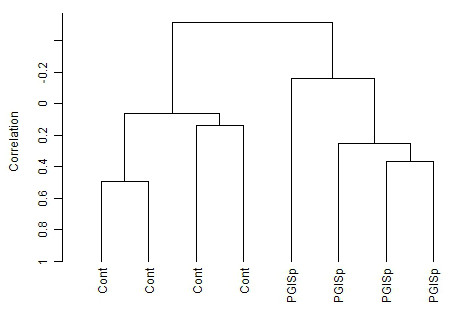
**Whole genome expression profiling of affected proteoglycan-induced spondylitis (PGISp) mouse vertebrae**. Unsupervised clustering using centred correlation and complete linkage shows expression profiles clearly delineate between control and PGISp spines.

Having established that excess cartilage and bone-like tissue is laid down in this spondylitis model we then went on to investigate the underlying molecular changes that might underpin the tissue changes. We undertook microarray-based whole-genome expression profiling in spines from week-12 affected PGISp or control mice that had not been injected with PG extract, to define the gene expression changes associated with joint remodelling. As might be expected with such marked tissue changes, unsupervised clustering distinguished all samples from affected mice and unaffected controls (Figure 4). A class comparison analysis, using a multivariate permutation test providing 80% confidence that the false discovery rate was less than 10%, identified 656 differentially expressed genes. Of these, 125 were increased > 1.5-fold and 46 decreased > 1.5-fold. To identify the pathway changes that might underlie the tissue changes we undertook GO analysis. Table 1 shows that a number of inflammatory and immune pathways are upregulated, as would be expected in an inflammatory arthritis model. Underlying these pathway changes was upregulation in a number of inflammatory genes (all *P *< 0.01) (Table 2). Tissue remodelling enzymes were upregulated such as *matrix metallopeptidase 3 *(*Mmp3*) (5.3-fold) and *Mmp13 *(2.3-fold), and *tissue inhibitor of metalloproteinase 1 *(*TIMP1*) (3.5-fold), as were components of the IL1 signalling network, *Il1b *(1.6-fold) and its receptor *Il1r2 *(1.3-fold). Other SpA-associated genes were also altered including *Il28ra *(1.2-fold) and the gene encoding TNF receptor type I, *tumor necrosis factor receptor superfamily, member 1a *(*Tnfrsf1a*) (1.2-fold). Elevated inflammatory signalling was also indicated by significant increases in both *signal transducer and activator of transcription 1 *(*Stat1*) (1.2-fold) *and Stat3 *(1.2-fold).

**Table 1:** Gene ontology (GO) analysis of genes differentially expressed in control and proteoglycan-induced spondylitis (PGISp) mouse spines

GO category	GO term	Number of genes	LS permutation *P*-value	KS permutation *P*-value	Efron-Tibshirani's GSA test *P*-value
**GO:0002252**	immune effector process	225	0.00001	0.00196	< 0.005 (+)
**GO:0002376**	immune system process	995	0.00001	0.00002	0.005 (+)
**GO:0002444**	myeloid leukocyte mediated immunity	18	0.00001	0.00179	0.005 (+)
**GO:0002682**	regulation of immune system process	374	0.00001	0.01936	0.005 (+)
**GO:0002684**	positive regulation of immune system process	255	0.00001	0.00417	0.005 (+)
**GO:0006954**	inflammatory response	293	0.00001	0.00007	< 0.005 (+)
**GO:0006955**	immune response	562	0.00001	0.00001	0.005 (+)
**GO:0032963**	collagen metabolic process	30	0.00007	0.01778	0.005 (+)
	Osteoblast genes	16	0.00105	0.00005	0.02 (+)

Gene expression levels in a number of inflammatory pathways, as well as an osteoblast gene set are altered in PGISp spine samples. LS, log summary; KS, Kolmogorov-Smirnov summary; GSA, gene set analysis.

**Table 2:** Inflammation and osteoblast-associated genes over-expressed in proteoglycan-induced spondylitis (PGISp) spines

Gene	Fold change PGISp/Control	*P*-value	Gene symbol
*Matrix metallopeptidase 3*	5.29	3.50E-05	*Mmp3*
*Matrix metallopeptidase 13*	2.26	0.0086	*Mmp13*
*Tissue inhibitor of metalloproteinase 1*	3.48	0.0003	*Timp1*
*Interleukin 1, beta*	1.57	0.0087	*Il1b*
*Interleukin 1 receptor, type II*	1.28	0.0000	*Il1r2*
*interleukin 28 receptor alpha*	1.18	0.0068	*Il28ra*
*Signal transducer and activator of transcription 1*	1.18	0.0150	*Stat1*
*Signal transducer and activator of transcription 3*	1.21	0.0039	*Stat3*
*Bone sialoprotein*	2.62	0.0001	*Ibsp*
*Collagen Type I*	2.51	0.0005	*Col1a1*
*Collagen Type III*	2.42	0.0004	*Col3a1*
*Osteocalcin*	1.50	0.0297	*Bglap1*

We also looked for changes indicative of osteoproliferative activity. We generated an osteoblast gene list composed of well-characterised osteoblast-associated genes and used it to undertake a GO analysis. This showed a highly significant upregulation in this gene set (Table 1) with *Col1a1 *(2.5-fold), *bone sialoprotein *(*Ibsp*) (2.6-fold), *osteonectin *(*Sparc*), (1.6-fold) and *osteocalcin *(BGlap1) (1.5-fold) all upregulated (all *P *< 0.03) (Table 2).

**Figure 5:**
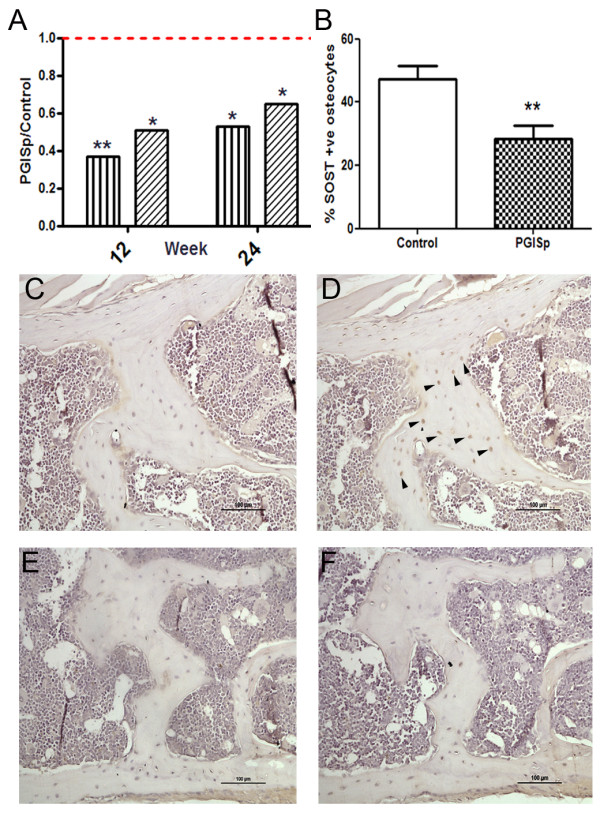
**Both Dickkopf-1 **(**DKK1) and sclerostin (SOST) are downregulated in proteoglycan-induced spondylitis (PGISp)-affected mouse spines**. (**A**) At week 12 and 24 both SOST and DKK1 expression are significantly downregulated in PGISp-affected spines, as shown by qPCR. Data are shown as Sost (vertical bars) or Dkk1 (diagonal bars) expression normalised to β-actin and expressed as the ratio of PGISp/Control. The dashed red line represents a ratio of 1, that is, no change in expression. Downregulation of SOST at the protein level was confirmed by immunohistochemistry. (**B**) The percentage of SOST-positive osteocytes is significantly reduced in the PGISp spine and pelvis. (**C**-**F**) Representative images demonstrating numbers of SOST+ve osteocytes in PGISp spines. Osteocyte-containing lacunae staining positive for SOST (**D**) are common in control bone but less evident in PGISp-affected samples (**F**). Isotype stained controls are also included (**C**,**E**). *P < 0.05, ** *P *< 0.01, PGISp vs. control.

As well as changes to bone matrix proteins we also investigated whether key bone regulatory genes were also altered that might be driving the osteoproliferative response. Using qPCR, we therefore specifically measured expression of the bone-associated Wnt inhibitors *Sost *and *Dkk1*, of which the expression levels are too low to be picked up by microarray. At both 12 and 24 weeks *Sost *(0.4-fold, *P *< 0.01 and 0.5-fold, *P *< 0.05, respectively) and *Dkk1 *(0.5-fold, *P *< 0.05 and 0.6-fold, *P *< 0.05, respectively) were significantly downregulated in PGISp-affected spines (Figure 5A). Downregulation of Sost in osteocytes within the vertebrae could also be seen at protein level (Figure 5B, C).

## Discussion

The PGISp model is a well-established mouse model of arthritis that exhibits both axial and peripheral inflammation [28] and has previously been presented as a model of spondylitis [13]. This further characterisation of axial disease pathology and underlying molecular changes in the Wnt signalling pathway further establish PGISp as a good model of the bone formation arising from joint inflammation typical of AS.

Axial disease in the PGISp mice was initiated with entheseal inflammation at the periphery of the vertebral and sacral joints. These inflammatory cells infiltrated the joint intervertebral area generating an invasive pannus, which destroys the IVD. This is then followed by proliferation of mesenchymal cells and deposition of a collagen/proteoglycan-rich matrix, which can eventually mineralise leading to ankylosis. Given this is the typical disease progression in AS [29], these observations establish the PGISp model as highly appropriate for investigation of AS aetiology. Initial inflammation occurs in axial insertion sites, such as the spinal and sacroiliac ligament attachments or sites of attachment of the outer fibres of the annulus fibrosus of the anterior vertebral disc. This initial enthesitis progresses and often a destructive spondylodiscitis is seen [30,31], as well as extensive cartilage loss preceding the pathological bone formation that underlies the joint fusion seen in advanced cases.

Whether the initial inflammation directly links to the subsequent osteoproliferative stages is subject to significant debate. There is considerable evidence that syndesmophytes develop at sites where previous inflammation has been observed [4]. Exactly how inflammation and bone formation are linked is not clear, with studies demonstrating that inhibition of inflammation does not affect radiographic progression in either human [32] or mouse [33] studies. However, it has also been shown in longitudinal human imaging studies that the presence of inflammatory lesions can predict the future development of syndesmophytes [34]. Our present study indicates the damage is certainly triggered by an initial inflammatory insult but there is little evidence of inflammatory cells remaining at sites where an extensive osteoproliferative response has occurred.

The study described here has further detailed the structural changes occurring at axial disease sites in the PGISp model and identified some of the molecular mechanisms that contribute to the disease process. IVD destruction follows the initial inflammation with extensive cell proliferation and excessive tissue formation then becoming evident. The nucleus pulposus is PG-rich, whereas the annulus fibrosus stains less strongly [35]. The matrix laid down in place of the destroyed disc in this model shows a different makeup, being rich in unmineralised PG, except at the periphery where collagen appears to comprise a significant component of the matrix. At this point, staining for unmineralised PG is negative, which might indicate a transition from a fibrocartilage-based matrix to immature bone. OCN staining is only present at the outer periphery of this matrix, indicating that this matrix has not yet developed into mature bone. It is clear that the model exhibits a strong anabolic response to the initial inflammatory insult.

We also sought to investigate the molecular changes underlying the progression through inflammation to bone formation using whole-genome expression analysis. Inflammatory as well as both matrix catabolic and anabolic pathways were altered. Elements of both the IL-1 and TNF pathways were upregulated. Both these pathways have been associated with AS through genetic studies [36,37]. IL28ra has been associated with psoriasis, a condition frequently co-existing in AS patients [38]. Stat1 and Stat3 mediate T_H_1 and IL-17-associated signalling respectively, with both cell types thought to play a role in the PGISp model [39,40] and have been implicated in SpA [41,42]. Elevated expression in these genes might reflect the increased T_H_1 and IL-17-expressing cell activity seen in these mice. *STAT3 *has also been shown to be associated with human AS [43]. These molecular disease patterns further support that the PGISp model replicates not only cellular changes in AS but also but also molecular patterns as well as inflammation polarization.

Mirroring the destructive nature of the early disease, stage changes in matrix remodelling factor expression levels were seen. MMP3, a stromelysin, and MMP13, a collagenase, key enzymes in extracellular matrix remodelling, which have both been shown to be elevated in AS as well as animal models [34,44,45], were both strongly upregulated. The strong matrix formation response was also reflected with marked upregulation of the key bone matrix components Col1, bone sialoprotein and OCN, as well as a number of other extracellular matrix-associated genes.

These gene expression changes mirror the tissue alterations seen in the joint but of further interest, particularly from a potential therapeutic point of view, are the molecules driving these tissue changes. The Wnt signalling pathway has been established as a key regulatory pathway for the bone-forming cells, osteoblasts, with SOST and DKK1 key inhibitors of Wnt signalling that are either specific to, or highly enriched in cells of the osteoblast lineage, respectively [14]. Reduced DKK-1 [16] and SOST [46] levels have been reported in AS patients. Blockade of DKK-1 has also been shown to drive ankylosis in a TNF-over-expressing mouse model of spondylitis [17]. Our data support this proposed elevated Wnt signalling in spondylitis with markedly decreased levels of the Wnt inhibitors DKK1 and SOST, the first such demonstration in a mouse model of SpA or AS.

## Conclusions

This study has demonstrated dysregulation of Wnt signalling in a mouse model of AS displaying excessive tissue formation, underlying syndesmophyte formation and ankylosis. It is likely in part that this dysregulation contributes to the osteoproliferation and supports targeting Wnt signalling therapeutically in AS. However, the key molecule(s) controlling the switch from inflammation to bone formation still require elucidation. Such molecules could provide excellent targets to develop targeted drugs to control the currently untreatable excessive bone formation leading to debilitating joint fusion.

## Abbreviations

ANKENT: ankylosing enthesopathy; AS: ankylosing spondylitis; β-cat: β-catenin; ColI: collagen type I; DKK-1: Dickkopf-1; EDTA: ethylenediaminetetraacetic acid; Fzd: Frizzled; GO: gene ontology; GSA: gene set analysis; GSK: glycogen synthase kinase; H&E: haematoxylin and eosin; HLA: human leukocyte antigen; IHC: immunohistochemistry; IL: interleukin; IVD: intervertebral disc; LRP: low-density lipoprotein receptor-related protein; MMP: matrix metalloproteinase; MRI: magnetic resonance imaging; OCN: osteocalcin; PC: proteoglycan; PG: proteoglycan; PGISp: proteoglycan-induced spondylitis mouse; qPCR: quantitative real-time reverse-transcription PCR; RA: rheumatoid arthritis; SOST: sclerostin; TNF: tumour necrosis factor; VST: variance stabilization transformation.

## Competing interests

The authors have no competing interests.

## Authors' contributions

KRH generated and analysed data and edited the manuscript. ARP provided technical expertise, analysed data and wrote the manuscript. RD generated and analysed data and edited the manuscript. HWT generated and analysed data and edited the manuscript. TTG provided technical expertise, provided reagents and edited the manuscript. MAB conceived the study and wrote the manuscript. GPT conceived and designed the study, analysed data and wrote the manuscript. All authors read and approved the manuscript for publication.
